# Predictors of family caregiving burden of persons with schizophrenia with and without transition of primary caregivers from 1994 to 2015 in rural China

**DOI:** 10.1192/bjo.2022.45

**Published:** 2022-04-01

**Authors:** Man-Man Peng, Zhiying Ma, She-Ying Chen, Wei Luo, Shi-Hui Hu, Xin Yang, Bo Liu, Cecilia Lai-Wan Chan, Mao-Sheng Ran

**Affiliations:** Institute of Advanced Studies in Humanities and Social Sciences, Beijing Normal University at Zhuhai, Zhuhai, China; Crown Family School of Social Work, Policy, and Practice, The University of Chicago, Chicago, Illinois, USA; Center for Social Work Study, Institute on Chinese Culture, Health and Public Welfare, Tsinghua University, China; Xinjin Second People's Hospital, Xinjin, Chengdu, Sichuan, China; Chengdu Mental Health Center, Chengdu, Sichuan, China; Guangyuan Mental Health Center, Guangyuan, Sichuan, China; Jingzhou Mental Health Center, Jingzhou, Hubei, China; Department of Social Work and Social Administration, University of Hong Kong, Hong Kong SAR, China; Department of Social Work and Social Administration, University of Hong Kong, Hong Kong SAR; and Mental Health Center, West China Hospital, Sichuan University, Chengdu, Sichuan, China

**Keywords:** Schizophrenia, primary caregivers, social functioning, caregiving burden, long-term care

## Abstract

**Background:**

Little is known about how sociodemographic and clinical factors affect the caregiving burden of persons with schizophrenia (PwSs) with transition in primary caregivers.

**Aims:**

This study aimed to examine the predictive effects of sociodemographic and clinical factors on the caregiving burden of PwSs with and without caregiver transition from 1994 to 2015 in rural China.

**Method:**

Using panel data, 206 dyads of PwSs and their primary caregivers were investigated in both 1994 and 2015. The generalised linear model approach was used to examine the predictive effects of sociodemographic factors, severity of symptoms and changes in social functioning on the caregiving burden with and without caregiver transition.

**Results:**

The percentages of families with and without caregiver transition were 38.8% and 61.2%, respectively. Among families without caregiver transition, a heavier burden was significantly related to a larger family size and more severe symptoms in PwSs. Deteriorated functioning of ‘social activities outside the household’ and improved functioning of ‘activity in the household’ were protective factors against a heavy caregiving burden. Among families with caregiver transition, younger age, improved marital functioning, deteriorated self-care functioning, and better functioning of ‘social interest or concern’ were significant risk factors for caregiving burden.

**Conclusions:**

The effects of sociodemographic and clinical correlates on the caregiving burden were different among families with and without caregiver transition. It is crucial to explore the caregiver arrangement of PwSs and the risk factors for burden over time, which will facilitate culture-specific family interventions, community-based mental health services and recovery.

As a chronic psychiatric disorder, schizophrenia is accompanied by disruptions in perception, cognition, emotions, behaviours and life functioning.^1,2^ The onset of schizophrenia typically starts in late adolescence or early adulthood, and the symptoms appear gradually.^3,4^ Frequent relapse and readmission to hospitals often afflict persons with schizophrenia (PwSs) and their families.^1^ Schizophrenia poses long-term challenges not only to the patient but also to his or her family, particularly the primary caregiver.

Previous research showed that caregiving burden remained significantly high among primary caregivers of PwSs with a deterioration in social functioning,^5,6^ worse self-care abilities^7^ and more care demands.^8^ The theory of transactional stress and coping can be applied to conceptualise the relationships between different patterns of stressors and caregiving burden.^9,10^ According to this theory, caregiving burden is defined as a transaction between a caregiver and the surroundings that is perceived by a caregiver as surpassing his or her available resources and being a threat to personal well-being.^11,12^ Family caregivers’ perceived burden is affected by factors from their internal world and external surroundings, such as the care recipients’ more severe symptoms and impaired social functioning.^2,13^ Prospective studies examining the associations between clinical characteristics and caregiving burden have documented inconsistent results. Specifically, some studies have found that increased severity of psychopathology and reduced social functioning predicted a heavier burden.^14,15^ Nevertheless, a recent longitudinal study reported that clinical changes in schizophrenic symptoms were not significantly related to long-term experiences of the caregiving burden.^13^ Although previous studies suggested the psychological consequences of impaired social functioning on caregivers,^2,16^ most of them were cross-sectional. Little is known about the effects of changes in patients’ social functioning over time on the caregiving burden throughout the illness trajectory.

The caregiving burden for PwSs is a complicated aspect of mental healthcare.^14^ In many societies, the original family is deemed the most significant social contact for a person.^17^ A high level of caregiving burden is prevalent among family caregivers of PwSs. For instance, a substantial number of studies have reported a high risk of psychological distress experienced by parent caregivers.^18–20^ Marriage or partnership was also greatly influenced by the illness, especially among cases in which the spouse caregivers resided with and cared for the PwSs for many years.^17^ Recent studies found that the psychological well-being of sibling caregivers was affected by a significant caregiving burden.^21,22^

Family structure and caregiving-related arrangements may change over time. Parents are usually expected to take a caregiver role throughout their child's long-term illness trajectory, particularly when the ill child does not have a partner.^23–25^ As parents get older, siblings might take over the caregiving responsibilities for their ill sister or brother from their parents.^22,26^ When transitioning to being the primary caregiver of a PwS, the new caregiver may be confronted with great challenges and need to adapt to the caregiving role.^27^ However, there has been limited research exploring the determinants of caregiving burden when caregiver transition occurs in a family of PwSs. In this study, caregiver transition was defined as a change of a PwS's primary caregiver from one type of family caregiver (e.g. a parent caregiver) at baseline to another type (e.g. a spouse caregiver) at follow-up. Caregiving burden may vary across different caregiving roles. For example, a recent empirical study reported that parent caregivers experience a significantly higher subjective burden than their spouse counterparts.^28^ Parent and spouse caregivers of PwSs may perceive the psychological burden differently.^29,30^ Nevertheless, few studies have been conducted to evaluate the longitudinal predictors of caregiving burden in families with and without caregiver transition.

The objective of this study was to evaluate the effects of changes in social functioning on the caregiving burden for PwS with and without caregiver transition over a period of 21 years (1994–2015). We hypothesised that among families of PwSs in rural Chinese communities: (a) the caregiving burden might be predicted by sociodemographic characteristics of PwSs differently in families with and without caregiver transition; (b) the caregiving burden might be predicted by the severity of symptoms of PwSs differently in families with and without caregiver transition; and (c) the caregiving burden might be predicted by changes in social functioning of PwSs differently in families with and without caregiver transition over 21 years.

## Method

### Data and procedure

This study employed panel data from the Chengdu Mental Health Project (CMHP), which has been conducted in Xinjin County, Chengdu, China since 1994.^4,31,32^ Six townships were randomly sampled from all 12 townships of Xinjin County.^33^ This study analysed data from two epidemiological surveys conducted in 1994 and 2015.^34^ The same research methods were used for the two epidemiological surveys, including the sampling method, screening procedures and diagnostic criteria for psychosis.^32^ Details of the procedures used for the longitudinal surveys, including sampling methods, have been described in our previous studies.^32,34^ Initially, to identify potential cases of PwSs, trained investigators (e.g. nurses, psychologists or social workers) used the Psychoses Screening Schedule to interview the heads of all households in the six townships.^2^ If the head of household could not attend the interview, another household resident (priority list: parent, spouse, offspring or other) was invited.^2^ Afterwards, the trained psychiatrists carried out a comprehensive general psychiatric interview for the potential patients via face-to-face interviews in hospitals. With the purpose of maintaining diagnostic reliability, a diagnosis of schizophrenia was determined by psychiatrists with over 5 years of clinical experience, and they used the same diagnostic criteria from the ICD-10^33^ in 1994 and 2015. After identifying PwSs, the family caregivers (parent, spouse, child, sibling or other relative) were invited to attend investigations to report caregiving-related information.

The care recipients in this study met the following inclusion criteria: (a) diagnosed with schizophrenia in both waves (1994 and 2015); and (b) completed both waves of the surveys (1994 and 2015). The exclusion criteria were as follows: (a) PwSs died before 2015; and (b) family caregivers had a diagnosis of mental disorders. The eligible caregivers (a) had kinship with PwSs, which could include being the PwS's spouse, parent, child, sibling, child-in-law or other collateral relatives; (b) were aged 15 years or above; and (c) had been caring for the patient for more than 6 months in the past year.^35^ Family caregivers who were also mentally disabled or unable to communicate well were excluded.^32,35^ Among the 510 PwSs identified in 1994, 250 were investigated 21 years later in2015. Excluding those who did not have family caregivers (*n* = 25), the analytic sample in this study included 225 dyads of PwSs and primary caregivers who had completed the questionnaires in both 1994 and 2015. Ethical approval for conducting the investigations was provided by the University Human Research Ethics Committee of the West China University of Medical Sciences in 1994 and by the University of Hong Kong in 2015. Informed consent with signatures was collected from all respondents at each stage of the surveys.

### Measurements

#### Clinical characteristics

##### Severity of symptoms (measured in 2015)

The Positive and Negative Syndrome Scale (PANSS)^36^ was used to measure the severity of symptoms in PwSs. The 30-item PANSS is a seven-point Likert scale (from 1 = no symptoms to 7 = the most severe symptoms), in which all items are divided into three dimensions: positive, negative and general psychopathology. A higher score on the PANSS represents a more severe degree of symptoms of schizophrenia.^36^ The PANSS (Chinese version) has been validated in the Chinese population.^37,38^ In our study, the Cronbach's alpha coefficient of the PANSS was 0.90.

##### Social functioning (time-varying)

The social functioning of PwSs was measured using the Chinese version of the Social Disability Screening Schedule (SDSS)^39^ in both 1994 and 2015. The SDSS is a ten-item scale used to assess the overall severity of dysfunction of PwSs, with a higher score indicating a poorer level of social functioning.^39,40^ The scale has been validated in Chinese populations.^41^ The Cronbach's alpha coefficient of the SDSS was 0.95, and the total score ranged from 0 to 20.

#### Caregiving burden

##### Caregiving burden (measured in 2015)

The Burden Scale for Family Caregivers – short (BSFC-s)^42^ was used as a self-report instrument to evaluate the subjective burden of the family caregivers. This scale examined the following ten aspects of the caregiving burden: reduced life satisfaction, physical exhaustion, wish to run away, depersonalisation, decreased standard of living, health affected by caregiving, caregiving reducing strength, conflicting demands, worry about the future and relationships with others affected.^42^ The responses to each question were rated on a four-point Likert scale, coded as 0 = strongly disagree, 1 = disagree, 2 = agree and 3 = strongly agree.^42,43^ A higher total BSFC-s score represents a greater degree of subjective burden in informal caregivers.^42^ The three-level classification system was used to interpret the BSFC-s score, with 0 to 4 points ranked as none to a mild degree of subjective burden, 5 to 14 points as a moderate level,and 15 to 20 points as a severe to very severe level.^44^ In this study, the internal consistency reliability of the Chinese BSFC-s was verified, with a Cronbach's alpha coefficient of 0.94.

#### Sociodemographic characteristics

Sociodemographic characteristics in this study included patients’ age, gender, marital status and education; and caregivers’ relationship to the patient, age, gender, family size and self-reported annual income.

### Statistical analysis

Prior to replacing missing values with imputation techniques, Little's missing completely at random (MCAR) test was performed. The results of the MCAR test were not statistically significant (χ^2^ = 615.055, d.f. = 571, *P* > 0.05), indicating that the data were probably missing at random. Therefore, missing values were replaced by an expectation maximisation technique as appropriate for MCAR data. Descriptive and frequency analyses were carried out to compare the differences between groups with and without caregiver transition. The paired-samples *t*-test was used to compare clinical characteristics between 1994 and 2015. Analysis of variance was used to estimate the mean differences in caregiving burden within the groups with different clinical characteristics. The generalised linear model approach was used with time-varying clinical characteristics as independent variables and the follow-up caregiving burden as a dependent variable. In the regression analysis models, the hypothetical predictors included baseline demographic variables, total duration of illness, follow-up severity of symptoms and time-varying clinical characteristics (i.e. changes in social functioning). Together with the duration of schizophrenia and the PANSS, the sociodemographic variables were included as covariates in the regression analysis to estimate the associations of changes in clinical characteristics with caregiving burden. After adjusting for the aforementioned variables, illness-related predictors (categorical variables) were entered into the regression models, where the ‘stable status’ subtypes served as the reference groups. The outcome variable was the follow-up caregiving burden. SPSS version 24.0 was used for statistical analysis.

## Results

### Demographic characteristics and caregiving-related information

Table 1 presents a comparison of demographic characteristics between baseline and follow-up. In the analytic sample, the PwSs consisted of 82 (39.8%) males and 124 (60.2%) females, with average ages of 39.7 years (s.d. = 11.96) at baseline and 60.8 years (s.d. = 12.00) at follow-up. More than half of the PwSs (78.2%) were married at baseline; 21 years later, over half of them (67.5%) were married and 15.5% were widowed (χ^2^ = 156.82, d.f. = 16, *P* < 0.001). The mean durations of schizophrenia were 10.5 years (s.d. = 9.96) in 1994 and 29.9 years (s.d. = 11.23) in 2015. At baseline, there were 133 (64.6%) male caregivers and 73 (35.4%) female caregivers, with an average age of 45.7 years (s.d. = 12.13). At follow-up, there were 162 (78.6%) male caregivers and 44 (21.4%) female caregivers, with an average age of 57.2 years (s.d. = 10.15). In 1994, 59.7% of the PwSs were cared for primarily by their spouses and 19.4% by their parents. In 2015, 63.1% were cared for primarily by their spouses, 22.8% by their adult children and 12.1% by their parents (χ^2^ = 106.3, d.f. = 9, *P* < 0.001). Based on the three-level classification system suggested by Pendergrass et al,^44^ in 2015, most of the families (74.3%) experienced the caregiving burden at a severe to very severe level, and 25.2% of the families perceived the caregiving burden to be at a moderate level. The median scores for family income per year were RMB 1000 yuan (≈160.55 USD) in 1994 and RMB 23666.7 yuan (≈3799.8 USD) in 2015. The median numbers of family members were 3.5 (interquartile range [IQR] = 0.5) and 3.0 (IQR = 0.5) in 1994 and 2015, respectively.

**Table 1:** Descriptive statistics of baseline and follow-up variables

Variables	1994 (*N*_1_ = 206)			2015 (*N*_2_ = 206)			*P*-value^a^
	Total	Male PwSs (*n* = 82)	Female PwSs (*n* = 124)	Total	Male PwSs (*n* = 82)	Female PwSs (*n* = 124)	
Patient characteristics							
Age, mean (s.d.)	39.74 (11.96)	36.06 (11.81)	42.18 (11.48)	60.77 (12.00)	57.35 (11.88)	63.03 (11.58)	*P* < 0.001
Marital status, *n* (%)							
Unmarried	32 (15.5)	24 (11.7)	8 (3.9)	20 (9.7)	19 (9.2)	1 (0.5)	*P* < 0.001
Married	161 (78.2)	52 (25.2)	109 (52.9)	139 (67.5)	44 (21.4)	95 (46.1)	
Divorced	6 (2.9)	4 (1.9)	2 (1.0)	13 (6.3)	10 (4.9)	3 (1.5)	
Widowed	5 (2.4)	0 (0.0)	5 (2.4)	32 (15.5)	9 (4.4)	23 (11.2)	
Other	2 (1.0)	2 (1.0)	0 (0.0)	2 (1.0)	0 (0)	2 (1.0)	
Education, *n* (%)							
No formal education or unknown	55 (26.7)	15 (7.3)	40 (19.4)	57 (27.7)	15 (7.3)	42 (20.4)	*P* < 0.001
Primary school	87 (42.2)	40 (19.4)	47 (22.8)	94 (45.6)	43 (20.9)	51 (24.8)	
Middle school	58 (28.2)	23 (11.2)	35 (17.0)	45 (21.8)	20 (9.7)	25 (12.1)	
High school and above	6 (2.9)	4 (1.9)	2 (1.0)	10 (4.9)	4 (1.9)	6 (2.9)	
Duration of schizophrenia, mean (s.d.)	10.50 (9.96)	9.15 (9.02)	11.4 (10.47)	29.91 (11.23)	29.23 (10.75)	30.35 (11.55)	*P* < 0.001
Family characteristics							
Caregiver's age, mean (s.d.)	45.71 (12.13)	45.54 (14.83)	45.81 (10.02)	57.23 (10.15)	56.95 (9.95)	57.42 (10.31)	*P* < 0.001
Gender, *n* (%)							
Male	133 (64.6)	23 (11.2)	110 (53.4)	162 (78.6)	58 (28.2)	104 (50.5)	0.226
Female	73 (35.4)	59 (28.6)	14 (6.8)	44 (21.4)	24 (11.7)	20 (9.7)	
Caregiver's relationship to the patient, *n* (%)							
Parent	40 (19.4)	29 (14.1)	11 (5.3)	25 (12.1)	20 (9.7)	5 (2.4)	*P* < 0.001
Spouse	123 (59.7)	38 (18.4)	85 (41.3)	130 (63.1)	38 (18.4)	92 (44.7)	
Adult child	12 (5.8)	4 (1.9)	8 (3.9)	47 (22.8)	20 (9.7)	27 (13.1)	
Sibling	0 (0.0)	0 (0.0)	0 (0.0)	4 (1.9)	4 (1.9)	0 (0.0)	
Others^b^	31 (15.0)	11 (5.3)	20 (9.7)	0 (0.0)	0 (0.0)	0 (0.0)	
Family size, median (IQR)	3.51 (0.50)	3.00 (0.50)	3.51 (0.50)	3.00 (0.50)	3.00 (0.50)	3.00 (0.50)	*P* < 0.01
Family income per year (RMB), median (IQR)	1000 (283.04)	1000 (195.54)	1095.54 (100)	23666.67 (12075)	24172.43 (8100)	24172.43 (2836.22)	*P* < 0.001
Family caregiving burden, *n* (%)^c^							
Level 1: none to mild (0–4)	−	−	−	1 (0.5)	0 (0)	1 (0.5)	−
Level 2: moderate (5–14)	−	−	−	52 (25.2)	20 (9.7)	32 (15.5)	
Level 3: severe to very severe (15–30)	−	−	−	153 (74.3)	62 (30.1)	91 (44.2)	

PwS, person with schizophrenia.

a. To compare the variables between 1994 and 2015, paired-sample *t*-tests were used for between-group differences of continuous variables; χ^2^-tests were used for between-group differences of categorical variables.

b. Others = other relatives, no caregiver, or unknown.

c. Data regarding caregiving burden were not collected in 1994.

### Severity of symptoms and caregiving burden across the groups by caregiver transition

Table 2 shows the characteristics of the two groups by caregiver transition and gender. In the whole sample, most of the PwSs (37.9%) were cared for by their husband in both waves, and 9.7% were cared for by their wife in both waves; 14.1% transitioned to adult–child caregiving from other types, whereas 14.1% transitioned to spousal caregiving. Mean BSFC-s scores ranged from 15.3 (s.d. = 6.40) to 20.4 (s.d. = 3.1) among different types of families, indicating that the families experienced the caregiving burden at a severe tovery severe level on average.^44^ Significant differences across thegroups were observed in terms of the caregiving burden (F = 2.540, d.f. = 6, *P* < 0.05). Specifically, the results of the least significance difference test showed that mean BSFC-s scores were significantly higher in the ‘parental caregiving in both waves’group compared with the ‘spousal caregiving in both waves’ group (*P* < 0.01).

**Table 2:** Mean differences in follow-up caregiving burden across the groups

Variables	FCGs (*N* = 206), *N* (%)		BSFC-s			
	Male FCGs (*n* = 162)	Female FCGs (*n* = 44)	M (s.d.)	F/mean difference	d.f./s.e.	*P*-value
Transition in primary caregivers				2.540	6	0.022
Parental caregiving in both waves	15 (7.3)	5 (2.4)	20.08 (5.410)	−3.577**	1.114	0.002
Spousal caregiving in both waves (ref.)	78 (37.9)	20 (9.7)	16.50 (4.073)	−	−	−
Adult-child caregiving in both waves	7 (3.4)	1 (0.5)	16.74 (5.095)	−0.235	1.669	0.888
Transition into parental caregiving	1 (0.5)	4 (1.9)	20.38 (3.076)	−3.877	2.081	0.064
Transition into spousal caregiving	29 (14.1)	3 (1.5)	16.17 (4.433)	0.330	0.924	0.722
Transition into adult-child caregiving	29 (14.1)	10 (4.9)	16.45 (5.084)	0.053	0.859	0.951
Transition into sibling caregiving	3 (1.5)	1 (0.5)	15.25 (6.397)	1.254	2.315	0.589

BSFC-s, Burden Scale for Family Caregivers – short; FCGs, family caregivers; ref., reference group, least significance difference test.

** *P* < 0.01.

### Predictors of family caregiving burden over 21 years

Table 3 presents the potential predictors of caregiving burden between families with versus without caregiver transition. Among families without caregiver transition, a heavier caregiving burden was significantly related to a larger family size (β = 0.615, s.e. = 0.268, *P* < 0.05) and more severe symptoms in PwSs (β = 0.094, s.e. = 0.027, *P* < 0.01). The results also indicated that a lower level of caregiving burden was significantly associated with deteriorated functioning in ‘social activities outside the household’ (β = −4.611, s.e. = 1.380, *P* < 0.01) and improved functioning in ‘activity in the household’ (β = −2.969, s.e. = 1.184, *P* < 0.05). Among families with caregiver transition, a higher degree of caregiving burden was found to be significantly associated with younger age of PwSs(β = −0.131, s.e. = 0.037, *P* < 0.001), improved ‘marital functioning’ (β = 2.574, s.e. = 1.081, *P* < 0.05), deteriorated self-care functioning of PwSs (β = 2.738, s.e. = 1.376, *P* < 0.05) and improved functioning in ‘social interest or concern’ of PwSs (β = −4.926, s.e. = 2.290, *P* < 0.05).

**Table 3:** Regression coefficients of the predictors of caregiving burden among families with versus without transition of primary caregiver

Variables	Without transition of primary caregiver		With transition of primary caregiver	
	B	s.e.	B	s.e.
Interpret	11.283***	2.9767	18.776***	3.0115
Control variables				
Male	1.329	.8182	−0.997	0.9249
Age	−0.067	.0395	−0.131***	0.0370
Married	−0.023	1.0969	−1.782	1.0896
Family size	0.615*	0.2680	0.401	0.4627
Household annual income	0.000	0.0003	−0.001	0.0011
Duration of schizophrenia	0.041	0.0337	0.037	0.0411
PANSS total	0.094**	0.0270	0.022	0.0382
Change in occupational functioning (ref. stable)				
Deteriorated	0.395	0.9107	2.307	1.3041
Improved	−0.522	1.0692	0.893	1.4296
Change in marital functioning (ref. stable)				
Deteriorated	−1.570	1.4195	0.079	1.6922
Improved	−0.641	1.0782	2.574*	1.0806
Change in parental functioning (ref. stable)				
Deteriorated	1.623	1.2771	−0.178	1.3727
Improved	0.408	1.2270	2.740	1.4699
Change in social withdrawal (ref. stable)				
Deteriorated	2.129	1.2513	3.502	2.3545
Improved	−0.378	1.1550	−0.612	1.8790
Change in social activities outside household (ref. stable)				
Deteriorated	−4.611**	1.3802	−4.201	2.3322
Improved	1.643	1.1000	−0.446	2.0886
Change in activity in household (ref. stable)				
Deteriorated	−0.620	1.2526	−0.402	1.8588
Improved	−2.969*	1.1841	−1.353	1.6648
Change in family role performance (ref. stable)				
Deteriorated	0.164	1.5310	1.159	1.7337
Improved	0.308	1.2181	−1.640	1.6875
Change in care of self (ref. stable)				
Deteriorated	−1.288	1.2944	2.738*	1.3761
Improved	0.560	1.1376	−2.241	1.5138
Change in social interest or concern (ref. stable)				
Deteriorated	1.376	1.6680	0.207	3.0471
Improved	1.800	1.7636	8.541*	3.4528
Change in responsibility and forward planning (ref. stable)				
Deteriorated	2.119	1.7721	−2.224	3.1168
Improved	−1.268	1.6452	−4.376	3.1439

Note: *P < 0.05, **P < 0.01, ***P < 0.001 (two-tailed). Dependent variable: caregiving burden (follow-up).

## Discussion

To the best of our knowledge, this is the first study to measure the predictive effects of changes in sociodemographic and clinical factors on the caregiving burden in family caregivers with versus without caregiver transition. This study contributes to the literature by emphasising the importance of caregiver transition and changes in the social functioning of PwSs over time when exploring the longitudinal determinants of caregiving burden in rural Chinese communities.

### Predictors of caregiving burden in families without caregiver transition

In this study, compared with the families with PwSs having spouses as primary caregivers in both waves, the caregiving burden was significantly higher in families with PwSs having parents as primary caregivers in both waves. This result is consistent with a recent cross-sectional study reporting that parent caregivers experience a significantly higher degree of subjective burden than spouse caregivers in rural China.^30^ This result shows that additional social support is particularly warranted to assist parent caregivers in accessing appropriate services and available resources in communities.^20,45^ A larger family size predicted a heavier caregiving burden among the families of PwSs without caregiver transition, consistent with previous studies.^46,47^ The results of this study indicate that larger families or those with parents as primary caregivers providing care to PwSs for a long period of time in rural Chinese communities are potentially more vulnerable and need more support.

In accordance with previous findings,^2,48–50^ the results of this study add evidence to the linkage between the severity of symptoms and caregiving burden among families without caregiver transition during long-term home care. Evidence shows that caring for a PwS with greater severity of illness may exacerbate the strain in families over time in rural Chinese communities. For instance, based on previous studies, families might experience a decline in economic status because of the higher expenditure on medication and lower labour capacity of both patients and their family caregivers due to the illness.^34,47,51^

The results of this study showed that deteriorated functioning of ‘social activities outside the household’ predicted a lower degree of caregiving burden among families without caregiver transition. This result is in contrast to previous research indicating that a lower level of social interest or social interaction was linked to a greater degree of caregiving burden.^52,53^ Given the cultural influence (e.g. superstitions) and social stigma in rural areas of China, this finding may be related to the possibilities that PwSs with decreased social interest may lessen caregivers’ long-term distress in terms of worrying about the trouble that a PwS may cause outside the household (e.g. on farmland or in other workplaces or communities) or the fear of being stigmatised by non-family members.^54–57^ These possibilities warrant further investigation. In our study, the improvement of PwSs with respect to ‘activity in the household’ was found to be a protective factor against a heavy caregiving burden. This could be explained by the possibility that these patients might be able to interact with other family members or even assist them with housework and farm work, which could to some extent release caregivers from a heavy workload and improve family relationships, thereby alleviating caregivers’ psychological burden.^55,58^

### Predictors of caregiving burden in families with caregiver transition

Building on previous cross-sectional studies,^47,55,57,59^ the present study further examined the association between patient age and caregiving burden, demonstrating that taking care of a younger PwS might predict a higher degree of caregiving burden in families with caregiver transition. This may be related to the psychological distress of caregivers resulting from a higher prevalence of disruptive behaviours^60,61^ and poor medication adherence^62^ among younger adults with schizophrenia.

Among families with caregiver transition from 1994 to 2015, improved marital functioning of the PwSs was found to predict a higher degree of caregiving burden in rural Chinese communities. It is plausible that after the PwSs married, in families where the primary caregiver of PwSs changed from other family members to their spouse, the new spouse caregivers were confronted with a high degree of psychological distress in terms of adopting the caregiving role.^27^ In addition, in contrast to the aforementioned result in families without caregiver transition, improved ‘social interest or concern’ functioning in the PwSs was found to be a risk factor for a heavier burden among families with caregiver transition, consistent with prior studies.^52,53^ This finding may be related to family caregivers’ concerns about PwSs’ safety^20^ or caregivers’ fear of being discriminated against by neighbours owing to stigma about schizophrenia^54,55^ when PwSs frequently interact with non-family members in the rural Chinese context. The results of this study also indicated that deterioration in the self-care functioning of PwSs predicted a heavier caregiving burden. This finding is congruent with previous studies showing that family caregivers of PwSs are more likely to perceive psychosocial burden when taking care of PwSs with more care needs or a lower level of independence.^52,53^ This finding might be related to the possibilities that (a) when primary caregivers change over time, the new caregivers might be more stressed if they are not familiar with the long-term illness status of a PwS without basic self-care abilities; (b) as PwSs and their caregivers get older, issues regarding who will continue to take care of these patients and where the patients will stay after the caregivers pass away may become important concerns among the majority of family caregivers.^63^ Thus, the current findings suggest the importance of having family intervention programmes to help patients learn how to take care of themselves and to support new caregivers in learning how to familiarise themselves with patient self-care needs.

### Limitations

Several limitations of the present study are worth noting. First, given the main focus on the change in social functioning and predictors of the later caregiving burden, we did not repeatedly examine the caregiving burden at baseline and follow-up. Baseline symptom information was not collected. Thus, the current findings were limited to interpreting the covariation of associations between changes in the caregiving burden and changes in clinical correlates.^64^ Future studies are warranted to further assess the differences in family caregiving burden and its clinical correlates (including social functioning and severity of symptoms) at different stages throughout the trajectory of the illness. Second, when measuring caregiver transition, this study only compared the differences between caregiving statuses in 1994 and 2015. We acknowledged that fluctuations regrading detailed arrangements of primary family caregivers were not observed in the present study. Third, the small sample sizes of several subgroups, including the ‘adult-child caregiving in both waves’, ‘transition into parental caregiving’, and ‘transition into sibling caregiving’ subgroups, limited the transferability of the findings of this study. In addition, the risk of sample bias should be noted, given the high percentage of female patients in this study, which was somewhat inconsistent with a nationwide population-based study reporting that schizophrenia is more prevalent among males than females.^65^ In addition, the unusual number of married patients probably reflects the high proportion of female patients. Fourth, the mental status of PwSs was measured using a single item by professionals. Comprehensive measures should be considered in future longitudinal studies to provide more items to uncover patients’ ever-changing illness status. Fifth, the generalisability of these findings was limited by the rural sample and the drop-out patient sample (e.g. death cases) during the long time interval in the study. Therefore, the findings might not be applicable to those residing in urban areas or during a long hospital stay. A sample combining both rural and urban residents is suggested for future investigations.

### Policy and practical implications

Despite the limitations, this study provides empirical evidence that the effects of changes in sociodemographic and clinical correlates on the caregiving burden were significantly different from those of transition in primary caregivers over 21 years in rural Chinese communities. These findings could inform future culture-specific interventions to work with different types of households with persons suffering from schizophrenia for long periods of time. Identifying determinants associated with the caregiving burden is essential in developing more appropriate strategies for family interventions and home-based services in families of PwSs.^66^ Based on the social context of rural China, primary health professionals (e.g. village doctors), social workers or family therapists should provide culture-specific suggestions to the primary caregivers of PwSs based on kinship types and the actual situation of caregiver transition, along with detailed guidance for improving the quality of family caregiving. For instance, based on our findings, effective interventions are particularly needed to improve patients’ interest, skills and functioning in daily household tasks and to help family caregivers arrange these tasks to facilitate patient participation.

Moreover, for social policy-making and public service provision in mental health, more targeted policies and mental health services could be designed to serve families who are experiencing a great burden of care in underdeveloped areas in China.^32,66,67^ For instance, medical expense waivers, anti-stigma interventions, ongoing expert consultation, or stress management training could be provided by the local government to serve the families of PwSs. The findings of this study highlighted the needs of more vulnerable groups, such as larger-sized families, patients with more severe symptoms, younger patients and patients with poor self-care functioning. Regular self-management training and rehabilitation programmes could be tailored to PwSs cared for at home for long periods of time to assist them in reintegrating into their communities.

## Data Availability

The data are not publicly available because they contain information that could compromise the privacy of research participants.
